# Grapevine phenolic compounds influence cell surface adhesion of *Xylella fastidiosa* and bind to lipopolysaccharide

**DOI:** 10.1371/journal.pone.0240101

**Published:** 2020-10-02

**Authors:** Steven A. Lee, Christopher M. Wallis, Elizabeth E. Rogers, Lindsey P. Burbank

**Affiliations:** 1 Crop Diseases, Pests and Genetics Research Unit, USDA, Agricultural Research Service, San Joaquin Valley Agricultural Sciences Center, Parlier, CA, United States of America; 2 Foreign Disease and Weed Science Research Unit, USDA, Agricultural Research Service, Frederick, MD, United States of America; Universidade do Minho, PORTUGAL

## Abstract

Bacterial phytopathogen *Xylella fastidiosa* specifically colonizes the plant vascular tissue through a complex process of cell adhesion, biofilm formation, and dispersive movement. Adaptation to the chemical environment of the xylem is essential for bacterial growth and progression of infection. Grapevine xylem sap contains a range of plant secondary metabolites such as phenolics, which fluctuate in response to pathogen infection and plant physiological state. Phenolic compounds are often involved in host-pathogen interactions and influence infection dynamics through signaling activity, antimicrobial properties, and alteration of bacterial phenotypes. The effect of biologically relevant concentrations of phenolic compounds coumaric acid, gallic acid, epicatechin, and resveratrol on growth of *X*. *fastidiosa* was assessed in vitro. None of these compounds inhibited bacterial growth, but epicatechin and gallic acid reduced cell-surface adhesion. Cell-cell aggregation decreased with resveratrol treatment, but the other phenolic compounds tested had minimal effect on aggregation. Expression of attachment (*xadA*) and aggregation (*fimA*) related genes were altered by presence of the phenolic compounds, consistent with observed phenotypes. All four of the phenolic compounds bound to purified *X*. *fastidiosa* lipopolysaccharide (LPS), a major cell-surface component. Information regarding the impact of chemical environment on pathogen colonization in plants is important for understanding the infection process and factors associated with host susceptibility.

## Introduction

*Xylella fastidiosa* is a gram-negative bacterium and the causal agent of Pierce’s disease in grapevines, as well as numerous other leaf scorch diseases [1]. Restricted to the xylem tissue of plants, *X*. *fastidiosa* is transmitted to grapevine by xylem-feeding insects, including sharpshooters, leafhoppers, and spittlebugs [2,3]. The transition between colonization of the insect vector and plant host is a highly regulated process, which relies on quorum sensing to transition between an adhesive biofilm state and systemic motility dependent on type IV pili [4,5]. Cell-cell aggregation mediated by fimbrial adhesins is an essential part of early biofilm formation in *X*. *fastidiosa* [6,7]. Encoded by the *fim* operon (*fimACDEF*, *edcD*) which is expressed in planta, fimbrial type I pili are located at the cell poles and are distinct from the type IV pili used for twitching motility [7–9]. Binding of *X*. *fastidiosa* to solid surfaces also requires outer membrane adhesin proteins including XadA and hemagglutinin proteins HxfA and HxfB [7,10]. In planta, adhesion to xylem walls is modulated by formation of outer membrane vesicles which block immediate binding of bacterial cells. This process promotes dispersive movement throughout the vascular tissue prior to initiation of the adhesive phenotype important for insect acquisition which occurs when bacterial cells reach high density [11].

In addition to cell-density regulated changes, *X*. *fastidiosa* adaptation to the plant environment is enabled by evasion of initial recognition by the host, and protection against reactive oxygen species produced by plant metabolism and defense responses [12,13]. Lipopolysaccharide (LPS) is one of the prominent bacterial cell surface components that often triggers immune recognition by plant cells [14]. In *X*. *fastidiosa*, the external O-antigen portion of the LPS molecule affects cell-surface adhesion dynamics and biofilm formation, in addition to playing an important role in delaying immune recognition by the plant [13,15].

The chemical environment in plant tissues contributes to both physical adhesion interactions, and bacterial gene expression. Accordingly, plant metabolites and disease-induced changes in chemical environment in the xylem can have a significant impact on virulence associated phenotypes. Plants infected with *X*. *fastidiosa* had elevated concentrations of calcium, which promoted cell-cell aggregation and biofilm formation, and increased surface adhesion forces [16,17]. In a study conducted in olive trees, several lipid compounds produced by trees infected with *X*. *fastidiosa* inhibited biofilm formation and increased planktonic cell growth [18]. Differences in plant chemistry are also associated with *X*. *fastidiosa* resistant versus susceptible grapevine and olive varieties [19–21].

Xylem sap of grapevine (*Vitis* sp.) although relatively nutrient poor, contains low concentrations of mineral ions, amino acids, and secondary metabolites, which change according to disease status, cultivar, and plant growth conditions [22–24]. Secondary metabolites found in grapevine xylem sap include phenolic compounds such as stilbenoids, tannins, catechins, and coumaric acid derivatives [23]. Many of these phenolic compounds are associated with plant disease and host defense responses. The stilbenoid compound resveratrol is well-studied for its antioxidant properties [25], and also has a role in grapevine defense against fungal pathogens such as *Botrytis cinerea*, possibly through direct inhibition of pathogen growth [26]. Although present in xylem sap at lower concentrations than in other plant tissues, resveratrol is well studied in plant disease response. High amounts of resveratrol are produced during grapevine trunk disease caused by necrotrophic fungal pathogens, in addition to other phenolics such as epicatechin, and gallic acid [27,28]. Gallic acid accumulation in grape callus tissue is also associated with resistance to downy mildew (*Plasmopara viticola*) [29]. Phenolic compounds are equally important in bacterial-plant interactions, both in establishing symbioses and in pathogenesis. In *Rhizobium* nodule formation and in *Agrobacterium* pathogenesis, phenolics act as signaling molecules that alter bacterial gene expression [30]. On the plant side, in *Brassica* species the phenolic compound p-coumaric acid induces plant resistance to *Xanthomonas campestris* through the jasmonic acid signaling pathway [31].

Total concentration of phenolic compounds in grapevine xylem sap increases in response to *X*. *fastidiosa* infection, but consequences for the pathogen are not known [22]. Previous work investigating the effects of phenolic compounds including resveratrol, catechin, coumaric acid, and gallic acid on *X*. *fastidiosa* growth in vitro focused on antimicrobial properties of the chemicals in high doses [32]. As the naturally occurring concentrations of these chemicals in grapevine xylem sap are well below the level required for direct inhibition of bacterial growth, the role of phenolics in *X*. *fastidiosa*-grapevine interactions is likely more complex [22,32]. In this study we evaluate the effect of resveratrol, epicatechin, gallic acid, and coumaric acid, in concentrations that would naturally occur in grapevine xylem sap, on *X*. *fastidiosa* growth, surface adhesion, and cell-cell aggregation.

## Results

### Phenolic compounds inhibit cell-surface attachment and aggregation

**Fig 1:**
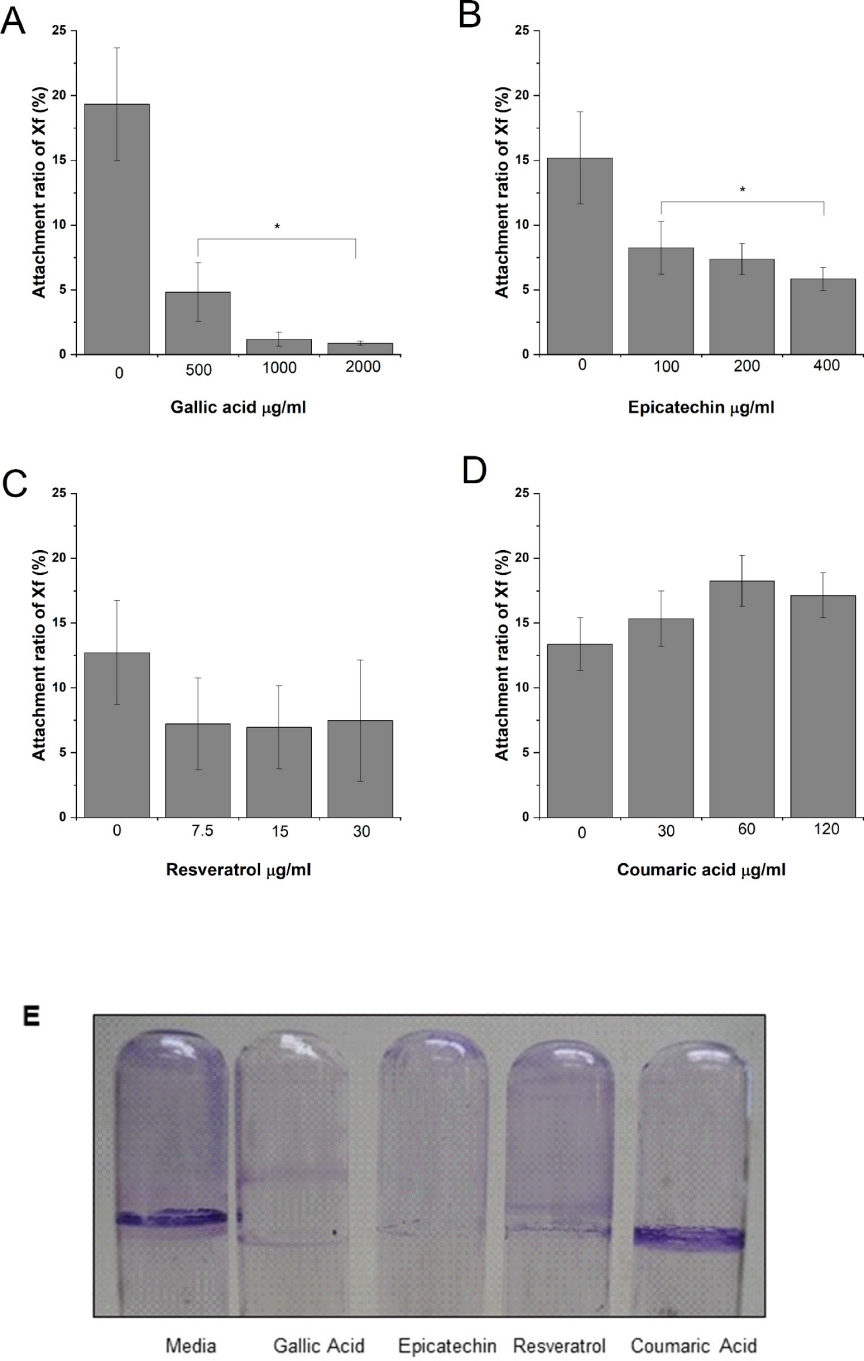
Gallic acid and epicatechin inhibit cell surface attachment. Quantitative cell-surface attachment of *X*. *fastidiosa* cultivated in liquid medium supplemented with GA (gallic acid, A), EP (epicatechin, B), RES (resveratrol, C), and CA (coumaric acid, D). Biofilm formation of cells grown with different treatments in polystyrene tubes (E). Data are expressed as mean ± standard deviation of three independent experiments (*n* = 8 replicates per experiment). Asterisk indicates significant differences (p-value < 0.005) in attachment ratio as determined by One-way ANOVA followed by Dunnett’s test comparing treatments to the media control.

**Fig 2:**
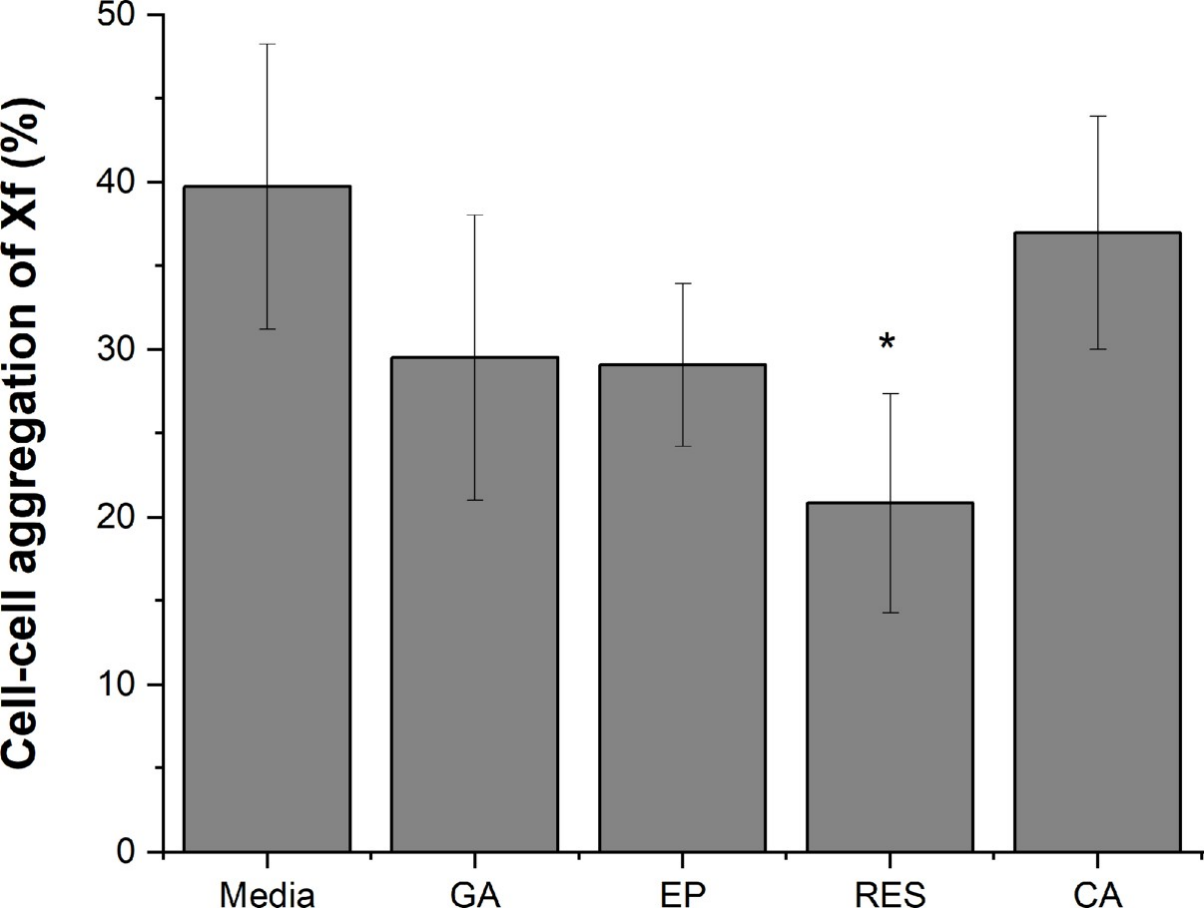
Resveratrol reduces cell-cell aggregation of *X*. *fastidiosa*. Percent cell–cell attachment of cells treated with gallic acid (GA), epicatechin (EP), resveratrol (RES), or coumaric acid (CA) for 7 d. Media was supplemented with GA (1000 μg/ml), EP (200 μg/ml), RES (15 μg/ml), or CA (60 μg/ml). Data are expressed as mean ± standard deviation of two independent experiments (*n* = 6). Significant differences (p-value < 0.005) in attachment ratio were determined by One-way ANOVA followed by Dunnett’s test comparing treatments to the media control and are indicated by asterisk.

Cell-surface attachment of *X*. *fastidiosa* strain Temecula-1 was quantified by crystal violet staining after growth in liquid medium supplemented with gallic acid (500, 1000, 2000 μg/ml), epicatechin (100, 200, 400 μg/ml), resveratrol (7.5, 15, 30 μg/ml), and coumaric acid (30, 60, 120 μg/ml)(Fig 1). Cultures supplemented with gallic acid (Fig 1A) and epicatechin (Fig 1B) showed a dose dependent decrease in surface attachment while resveratrol (Fig 1C) and coumaric acid (Fig 1D) did not affect surface attachment. Bacterial growth was not inhibited by any of the phenolic compounds at these experimental concentrations. Biofilms formed by growth in polystyrene tubes produced similar results (Fig 1E), except that resveratrol-treated cells produced a less distinctive biofilm ring at the air-liquid interface. Cell-cell aggregation was significantly reduced in medium supplemented with resveratrol (Fig 2), but no change in aggregation was observed in medium supplemented with any of the other compounds.

### *Xylella fastidiosa* growth causes depletion of phenolic compounds from culture medium

**Fig 3:**
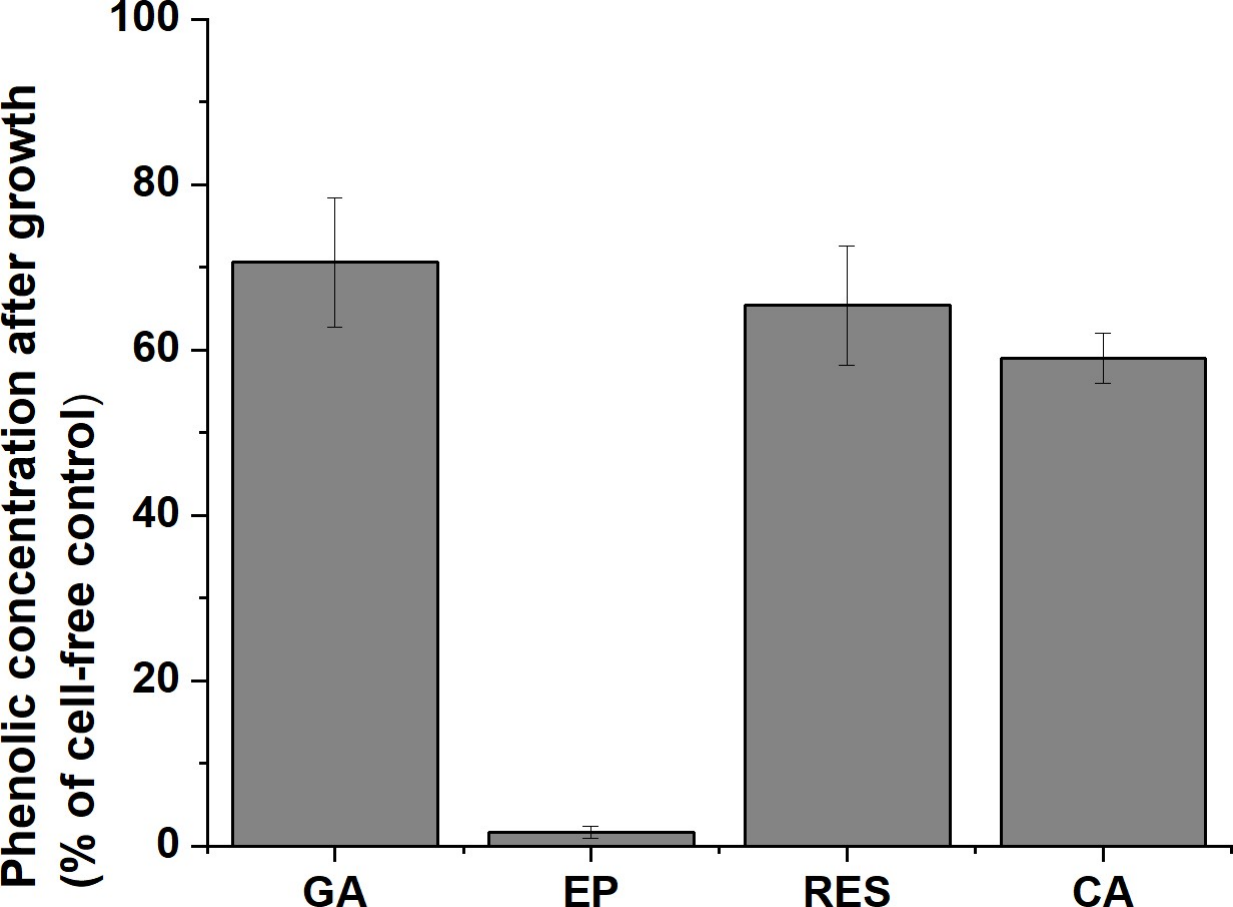
Depletion of phenolic compounds by growth of *X*. *fastidiosa*. Percent of gallic acid (GA), epicatechin (EP), resveratrol (RES), or coumaric acid (CA) remaining in cell-free supernatants after 7 days with X. fastidiosa cells. Media was supplemented with GA (1000 μg/ml), EP (200 μg/ml), RES (15 μg/ml), or CA (60 μg/ml). Bacterial cells were removed by filtering prior to measuring concentrations of gallic acid, epicatechin, resveratrol, or coumaric acid by HPLC. Data are expressed as means + standard deviation of three independent experiments (n = 3 replicates per experiment). Significant differences in the amount of compound remaining were compared by the Mann-Whitney Rank of Sum Test (T = 126, n = 9, p-value < 0.001) and significance is indicated by asterisk.

Concentration of gallic acid, epicatechin, resveratrol and coumaric acid in culture media were quantified by HPLC after growth of *X*. *fastidiosa* for seven days (Fig 3). Results showed that gallic acid was reduced by 30%, epicatechin by almost 98%, resveratrol by 24%, and coumaric acid by 41% (Fig 3). The cell-free supernatants and cell pellets were analyzed by LC-MS for presence of phenolic compounds that might have been break-down products or compound derivatives, but none were identified via these techniques.

**Fig 4:**
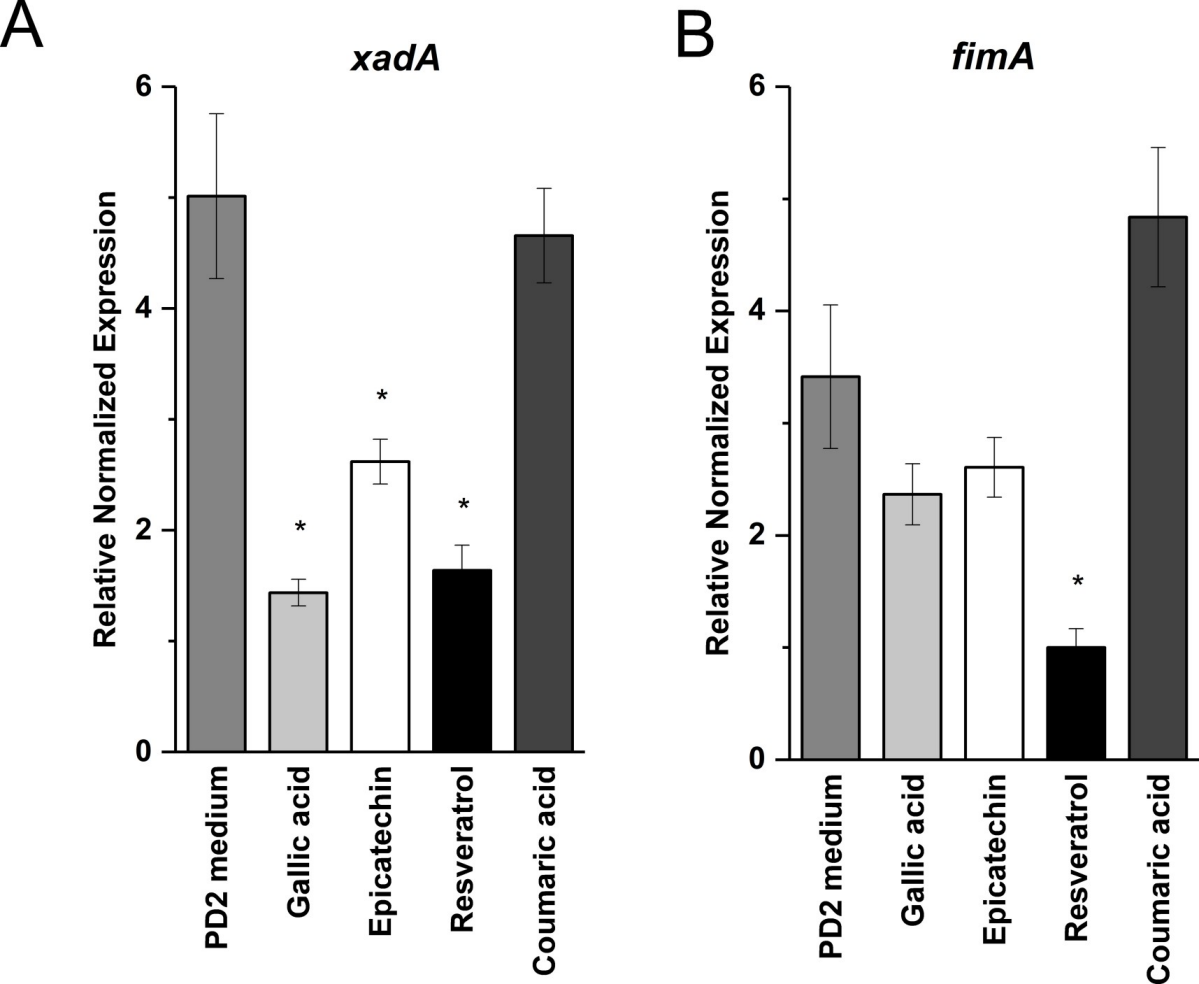
Reduced expression of adhesion-related genes in the presence of phenolic compounds. Expression of *xadA* (A) and *fimA* (B) in *X*. *fastidiosa* after 7 d of growth in the presence of gallic acid (1000 mg/ml), epicatechin (200 mg/ml), resveratrol (15 mg/ml), or coumaric acid (60 mg/ml). Data are expressed as means ± standard deviation of three independent experiments, each consisting of three biological replicates (*n* = 9). Significant differences (p-value < 0.05) in gene expression, relative fold change, standard error, and confidence intervals were all calculated by the CFX Manager software.

**Grapevine phenolics decrease expression of fimbrial and afimbrial adhesin genes.** Expression levels of *fimA* (Fimbrial subunit precursor A, PD0062) and *xadA* (Xanthomonas Adhesion, PD0731) were quantified by RT-qPCR in *X*. *fastidiosa* cultivated in liquid medium supplemented with phenolic compounds (Fig 4). Expression of *xadA* was significantly reduced in cells treated with gallic acid, epicatechin, and resveratrol (Fig 4A). Cells supplemented with resveratrol also showed significantly decreased expression levels of *fimA* (Fig 4B). Coumaric acid had no effect on expression of *fimA* or *xadA*.

### Grapevine phenolics bind to *X*. *fastidiosa* lipopolysacchride

**Fig 5:**
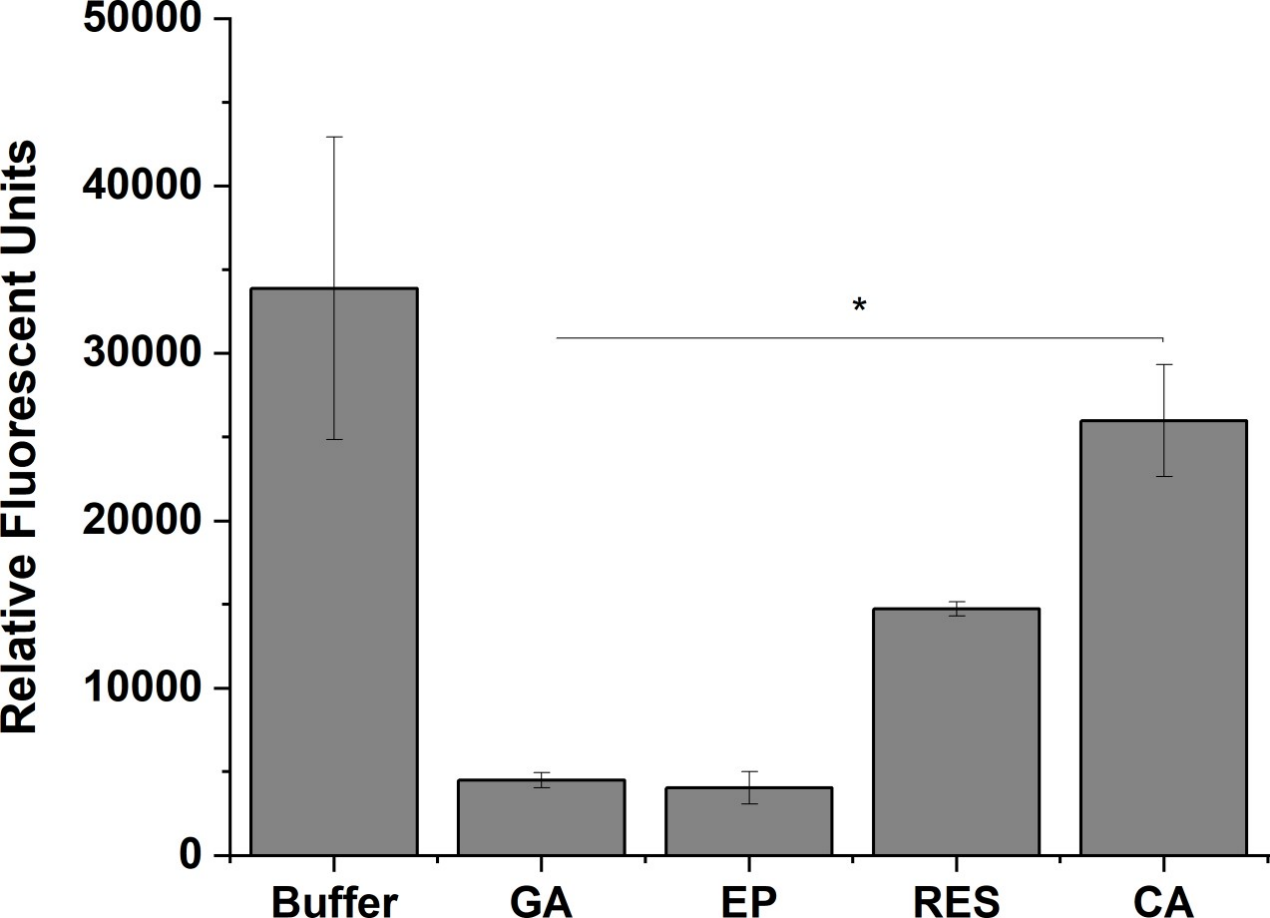
Phenolic compounds bind to *X*. *fastidiosa* lipopolysaccharide. Binding of compounds to LPS was quantified using the relative florescence of FITC-LPS bound to Polymixin B agarose beads after 1-hr incubation with buffer, gallic acid (1000 μg/ml), epicatechin (200 μg/ml), resveratrol (15 μg/ml), or coumaric acid (60 μg/ml). Data are expressed as means ± standard deviation of three independent experiments (n = 4 replicates per experiment). Significant differences (p-value < 0.001) in relative florescence determined by One-way ANOVA followed by Dunnett’s test comparing treatments to the buffer control are indicated by asterisk.

Gallic acid, epicatechin, resveratrol, and coumaric acid were analyzed for binding to *X*. *fastidiosa* lipopolysaccharide (LPS), a major component of the bacterial cell surface. Binding activity was quantified by a competitive binding assay using fluorescently labelled purified LPS from *X*. *fastidiosa* and polymixin B conjugated agarose beads [33]. All four of the phenolic compounds bound to *X*. *fastidiosa* LPS (Fig 5). Gallic acid and epicatechin showed the highest binding affinity as demonstrated by reduction in fluorescence. Coumaric acid showed the lowest binding capacity for LPS but still produced a significant reduction in fluorescence compared with the untreated control (Fig 5).

## Discussion

The role of phenolic compounds in cell adhesion and biofilm formation is well established for human pathogenic bacterial species [34], but limited information is available regarding the effects of these compounds on xylem-colonizing plant pathogens, despite the plant-based origins of these chemicals. Resveratrol and its oligomers inhibited biofilm formation in human pathogenic *E*. *coli* and *Pseudomonas aeruginosa* [35], and other stilbene compounds also have anti-biofilm activity against fungal pathogen *Candida albicans* [36]. Similarly, gallic acid disrupted adhesion and biofilm formation in *E*. *coli*, *P*. *aeruginosa*, *Staphylococcus aureus* and *Listeria monocytogenes* [37]. Tannins, a chemical class including catechin compounds, not only disrupted biofilm formation of both gram-negative and gram-positive bacterial pathogens, but also suppressed other virulence associated traits such as exopolysaccharide production and antibiotic resistance [34,38].

In this study, grapevine-associated phenolic compounds epicatechin, and gallic acid inhibited *X*. *fastidiosa* cell-surface attachment in vitro. Resveratrol at concentrations found in xylem sap did not significantly impact cell surface attachment overall, but reduced auto-aggregation which is essential for the early stages of biofilm formation in *X*. *fastidiosa*. Cells cultivated in the presence of resveratrol also did not form as much of a characteristic biofilm ring at the air-liquid interface, likely because deficiency in cell-cell aggregation interferes with proper biofilm structure formation [39]. The phenolic concentrations in this study were in the range of those found in grapevine xylem sap and were not high enough to inhibit bacterial growth. However, influence on attachment and aggregation phenotypes suggest an important role of phytochemicals in the *X*. *fastidiosa* infection process in grapevine. Not all phenolic compounds present in grapevine xylem sap showed this effect, as coumaric acid did not alter cell attachment or aggregation when present in biologically relevant concentrations. Similar phenotypic changes were seen with olive pathogenic strains of *X*. *fastidiosa* exposed to phytochemicals (lipid compounds) present in infected olive tissue, suggesting a broad role of plant produced compounds in progression of disease caused by this pathogen in different host plants [18].

Cell-surface attachment and cell-cell aggregation are part of the complex biofilm development process of *X*. *fastidiosa* that involves widespread transcriptional changes. In a previous study, *xadA*, encoding an outer membrane adhesin essential for *X*. *fastidiosa* surface attachment was expressed at higher levels in xylem sap from grapevine compared with citrus xylem sap suggesting gene regulation is influenced by chemical environment specific to plant species [40]. Expression of *xadA* in *X*. *fastidiosa* was reduced when growth medium was supplemented with gallic acid, epicatechin, or resveratrol, which may have contributed to the reduction in surface attachment in gallic acid and epicatechin treated cells. Although resveratrol treated cells did not show significantly reduced attachment, expression of *xadA* alone likely does not provide the whole picture. Other afimbrial adhesins such as the hemagglutinins are also important for *X*. *fastidiosa* surface attachment and could play a compensatory role under some growth conditions [10,41].

In addition to adhesins, initial stages of biofilm development in *X*. *fastidiosa* require cell-cell aggregation mediated by fimbrial type I pili. Expression of *fimA*, a fimbrial subunit protein essential for formation of type I pili, was significantly reduced when *X*. *fastidiosa* cultures were supplemented with resveratrol, but the effect was not observed when other phenolics were tested. This was consistent with the reduced aggregation phenotype observed in resveratrol treated *X*. *fastidiosa* cells. A similar decrease in expression of fimbrial production genes in *E*. *coli* was responsible for disruption of biofilm formation by resveratrol oligomers [35]. It is likely that phytochemicals in the plant environment impacted bacterial colonization in many ways depending on the specific chemical compound, as well as the timing and concentrations produced by the plant during disease progression.

In addition to altering gene expression, phenolic compounds impact physical surface interactions of pathogens with host cells [42], and both pathogenic and beneficial bacterial species can metabolize plant phenolics for growth [43–45]. In this study, resveratrol, epicatechin, gallic acid and coumaric acid were removed from the culture medium by growth of *X*. *fastidiosa*. Metabolic breakdown products or other compound derivatives were not identified via the LC-MS based analyses performed, either in culture supernatants or in cell cytoplasmic fractions, albeit future studies would be warranted to incorporate more advanced techniques to truly determine the fate of the phenolics removed from culture. That said, all four of the phenolic compounds bound to purified *X*. *fastidiosa* LPS, suggesting that a significant depletion from the growth medium may occur through direct binding to the bacterial cell surface. LPS in gram-negative bacteria makes up 75% of the outer membrane [46], and in *X*. *fastidiosa* is important for cell attachment, aggregation, and cell surface properties [15,47]. In human pathogenic *E*. *coli*, phenolic compounds derived from plant extracts (grape, cranberry, and tea) bind to LPS and block adhesion to the host cell surface [42]. LPS-phenolic binding interactions are strong enough that use of plant phenolic compounds has been proposed as a method of removing bacterial LPS from solutions [48]. In *X*. *fastidiosa* infection in grapevine, binding of phenolic compounds to LPS during xylem colonization may contribute to reduced adhesion in addition to gene expression changes.

Overall it is apparent that specific phenolic compounds present in grapevine xylem sap influence virulence associated phenotypes of *X*. *fastidiosa*, even when present at low concentrations. Further work is required to fully understand how this pathogen adapts to the chemical environment within the plant, and how these interactions are affected by host plant species, pathogen strain, other biotic or abiotic stress factors, level of host resistance, and stage of disease progression. Understanding how xylem chemistry alters bacterial infection dynamics is essential for development of novel strategies to mitigate *X*. *fastidiosa* damage in perennial crops when pathogen elimination is not feasible.

## Materials and methods

### Phenolic compounds for testing

Gallic acid, epicatechin, resveratrol and coumaric acid were obtained from Sigma-Aldrich Chemical Company (St. Louis, MO). Stock solutions of chemical compounds were freshly prepared by dissolving compounds in dimethyl sulfoxide (DMSO) at 50 mg/mL (gallic acid, epicatechin, and coumaric acid) or 30 mg/mL (resveratrol). Compounds were diluted to final experimental concentration using PD2 liquid medium. DMSO (2.0%) was added to all media controls and at this concentration does not have any impact on cell growth.

### Bacterial strain and culture conditions

The grape pathogenic strain of *X*. *fastidiosa* Temecula-1 [49] was used for all experiments. Bacteria were cultivated at 28°C on solid or in liquid PD2 media [50]. For PD2 solid medium 12 g/L of Gelzan (Sigma-Aldrich, St. Louis, MO) was used instead of agar. For each experimental replicate a fresh plate of *X*. *fastidiosa* was streaked from glycerol stocks stored at -80°C freezer and allowed to grow for 7 days prior to experiments.

### Quantification of bacterial growth and cell-surface attachment

Cell attachment was quantified using protocols previously described for *X*. *fastidiosa* attachment and biofilm development with slight modification [16,51]. *X*. *fastidiosa* cells were harvested from PD2 plates and suspended in liquid PD2. Cell suspensions were adjusted to a concentration of OD_600_ = 0.2 (approximately 2x10^6^ cfu/mL). Gallic acid, epicatechin, resveratrol and coumaric acid were diluted to 2x of final concentration in PD2 media. Aliquots of 100 μL of *X*. *fastidiosa* cells and 100 μL media containing gallic acid, epicatechin, resveratrol or coumaric acid were mixed in individual wells of 96-well polystyrene culture plates for a final concentration of 1x10^6^ cfu/mL. Final concentrations of phenolic compounds were: gallic acid, 500–2000 μg/ml; epicatechin, 100–400 μg/ml; resveratrol, 7.5–30 μg/ml; coumaric acid, 30–120 μg/ml). Cultures were incubated for 7 days at 28°C without shaking. After incubation, media containing planktonic cells were transferred to a new 96 well plate without disturbing the attached cells and absorbance of the transferred cells was quantified by OD_600_. Wells containing attached cells were washed 3 times with double-distilled water to remove any remaining planktonic cells. Attached cells were stained with 0.1% (w/v) crystal violet in water for 20 min at room temperature. Crystal violet was decanted, and wells were washed with double-distilled water 3 times. Crystal violet absorbed by the attached cells was eluted with 250 μL of 50% methanol/50% acetone and the eluent was quantified by measuring absorbance at 600nm [52]. Eight technical replicates were performed per experiment and experiments were repeated three times with separate cultures. The attachment ratio was determined by dividing the absorbance of the crystal violet by the optical density of the planktonic cell suspension [10].

### HPLC analysis of media components after bacterial growth

Spent media supernatants from planktonic cell samples were collected after culturing for 7 d and subjected to centrifugation at 4300 x g for 10 minutes. Supernatants were passed through a 0.22 μm filter prior to being placed in glass vials for high-performance liquid chromatography (HPLC) analyses as previously described [22]. In brief, a total of 50 μL of spent media was injected into a Shimadzu (Columbia, MD, USA) LC-20AD HPLC system equipped with a Sulpelco (Bellefonte, PA, USA) Ascentis C18 column (25cm by 4.6mm, 5μm pore size) and a Shimadzu SPD-20A photodiode array detector set at 280nm. A binary gradient was used proceeding from 95% solvent A [water with 0.2% v/v acetic acid (Sigma)] to 100% solvent B [methanol (Sigma) with 0.2% acetic acid] over 40 minutes. The peak for each compound was confirmed by running commercial standards obtained from Sigma-Aldrich, and a dilution series was run to convert peak areas into grams. A Shimadzu LCMS-2020 single-quad mass spectrometer in electron spray ionization mode attached to the HPLC system was used to further analyze potential breakdown productions by scanning ions from 150 to 1000MW in both positive and negative ionization modes [22].

### Gene expression analysis

RNA was extracted using the hot phenol extraction method described by Jahn et al [53]. Samples were treated with BaselineZero DNase (Illumina) following the manufacturers’ instructions to remove all genomic DNA, and RNA integrity was evaluated by gel electrophoresis. Purified total RNA was quantified using the QuantIT RNA kit (Life Technologies) plate reader protocol on an Infinite M1000 fluorescent plate reader (Tecan). Synthesis of cDNA was performed using 500 μg of purified total RNA and the iScript cDNA synthesis kit (BioRad). After reverse transcription, samples were diluted 1:10 in sterile dH_2_O and 2 μl was used as a template for qPCR using AB Fast SYBR Green (Life Technologies) master mix following the manufacturers’ recommendations. PCR primer sequences are listed in Table 1 for *xadA*, *fimA*, and reference gene *dnaQ*. PCR conditions are as follows: 95°C for 3 min, 30 cycles of 95°C for 3 s followed by 60°C for 30 s with fluorescence detection at the end of each cycle. PCR and fluorescence detection were performed on a BioRad CFX96 instrument. Three biological replicates were included for each of the treatments and the media control samples. RNA extraction and qPCR was repeated independently three times and presented as average relative gene expression +/- standard error of the mean. Gene expression, relative fold change, standard error, and confidence intervals were all calculated by the CFX Manager software (BioRad).

**Table 1:** List of primers used in this study.

Primer	Sequence	Source
xadA-qPCR-fwd	TCGGTCTTGCGCTTACAAGT	This study
xadA-qPCR-fwd	CACATGAGCCACCATGACCT	This study
fimA-qPCR-fwd	CGACAGAACCTGCACCATCA	This study
fimA-qPCR-rev	AGCTGAACGATACTGTCGGC	This study
dnaQ-qPCR-fwd	CGTTATCCGGGTCAGCGTAA	[54]
dnaQ-qPCR-rev	GTAACTGACGGTGGGCGTTA	[54]

### Quantification of cell-cell aggregation

*X*. *fastidiosa* cultures were incubated for 7 days in the presence of phenolic compounds using 15-mL polypropylene tubes. Cultures were left without any movement for 30 min at room temperature and the planktonic (non-aggregated) cells were removed with a pipette and measured by OD_600_, (OD_s_). Culture medium was then returned to the original tube and all aggregated cells were dispersed by pipetting. Turbidity of total cell culture was measured immediately after mixing by OD_600_, (OD_t_). Percent aggregated cells was calculated using the following formula = (OD_t_-OD_s_)/OD_t_-100 [55].

### Lipopolysaccharide extraction and binding assays

*X*. *fastidiosa* cells were cultivated on PD2 medium for 5 days at 28°C. Cells were harvested from multiple plates with 1x PBS, pooled in 50 mL tubes, and pelleted by centrifugation at 15000 rpm for 5 mins. Pellets were washed twice with 10 mL of 1xPBS and re-suspended at a concentration of OD_600_ = 1.2. Aliquots of 2 mL were used for LPS extraction using the LPS Extraction Kit by iNtRON biotechnology (Kyungki-Do, S. Korea) following the manufactures’ protocol. LPS was quantified using the Purpald Assay [56]. Extracted LPS was conjugated with fluorescein isothyiocyanate (FITC, Sigma) using the following protocol. LPS (10 mg) was incubated with 20 mg of FITC in 5.0 mL of 0.1 sodium borate buffer, pH 10.5, for 3 h at 37°C. Conjugates were dialyzed for 48 h against 0.15M NaCl to remove unincorporated dye. Concentration of FITC in the FITC-LPS was determined spectrophotometrically [33]. Conjugated FITC-LPS was incubated in the presence or absence of gallic acid (1000 μg/ml), epicatehcin (200 μg/ml), resveratrol (15 μg/ml), or coumaric acid (60 μg/ml) for 1 h at 25°C in the dark. Polymyxin B conjugated to agarose beads (Sigma-Aldrich) were added to reactions and incubated for 1 h at 25°C in the dark. Beads were centrifuged and washed 3 times with 250 μl of 0.05M Tris buffer to remove unbound FITC-LPS. Beads were re-suspended in 200 μl nuclease free water and added to a 96 well plate black round bottom plate. Fluorescence was measured using 435nm excitation and 535nm emission on an Infinite M1000 Pro plate reader (Tecan). Three independent assays were run with 4 replicates per treatment. Fluorescence values are represented as mean +/- standard deviation. One-way ANOVA followed by Dunnett’s comparison test was used to compare means of treatments to the media control.

## Supporting information

S1 File This file contains raw data used to produce figures. (XLSX)
